# Gunshot Wounds of the Knee-joint

**Published:** 1918-12

**Authors:** 


**Gunshot Wounds of the Knee-Joint with Septic Arthritis.** Major Arthur NEVE, R. A. M. C. *The Lancet*, Sept. 14, 1918.

No further evidence is now required to prove that most knee-joints, which have been perforated by bullets or severely wounded by shell fragments, with injury to the bone, may be saved by timely measures. Articles by Sir George Makins and Colonel Barling show that cases with septic arthritis, especially when associated with damage to the bone, stand in infinitely greater danger than the others.

The more recent convoys from France received at Dartford have shown many encouraging results obtained in France from early operation with incision of the wound, removal of foreign bodies, and primary suture. A year ago similar injuries would have arrived at the hospital in a highly septic condition.

Drainage : The chief question is how to obtain adequate posterior drainage, for the back of the joint is swathed in ligamentous and muscular structures, etc., and in close contact with the posterior ligament is the popliteal artery. Obviously any drainage tube behind the crucial ligament would risk hemorrhage. But postero-lateral incisions should be made to the synovial pouches behind the condyles, by the method of Labey adopted by Campbell and Gill. Anterior drainage is comparatively easy.

Major Neve has not had satisfactory results with long, transverse anterior incisions, nor does he favor partial excision with separation of the bones; though carried out in conjunction with the Carrel method he believes it promising.

Fullerton's method may be regarded as a method of insuring thorough drainage. In 1915 many surgeons were using normal saline, or weak iodin, or permanganate solutions; then came Dakin's hypochlorite. Continuous irrigation certainly saved some limbs, but surgeons could not fail to see that the solutions too often poured along the gutters without flushing the whole road, or reaching the joint pouches. That is where Carrel's technic is valuable, instilling the antiseptic into every recess, but Carrel is not very optimistic himself about the method as applied to developed sepsis of the knee.

The knee-joint figures for 1918, up to September, show a great improvement on the two previous years.

In the earliest stage of infective traumatic arthritis, an attempt should be made by tapping and distending the joint with some antiseptic, such as flavine or iodoform emulsion, to abort the sepsis. This is being successfully done in France.

